# Genome-wide association study of paediatric bacteraemia and sepsis

**DOI:** 10.1016/j.ebiom.2026.106320

**Published:** 2026-06-04

**Authors:** Dylan Lawless, Flavia Aurelia Hodel, Christian W. Thorball, Zhi Ming Xu, Alessandro Borghesi, Eric Giannoni, Johannes Trück, Martin Stocker, Klara M. Posfay-Barbe, Ulrich Heininger, Sara Bernhard-Stirnemann, Anita Niederer-Loher, Christian R. Kahlert, Giancarlo Natalucci, Christa Relly, Christoph Berger, Thomas Riedel, Christoph Aebi, Philipp Agyeman, Jacques Fellay, Luregn J. Schlapbach, Swiss Pediatric Sepsis Study Group, Christoph Aebi, Philipp K.A. Agyeman, Walter Bär, Christoph Berger, Sara Bernhard-Stirnemann, Eric Giannoni, Ulrich Heininger, Christian R. Kahlert, Gabriel Konetzny, Antonio Leone, Giancarlo Natalucci, Anita Niederer-Loher, Klara M. Posfay-Barbe, Christa Relly, Thomas Riedel, Luregn J. Schlapbach, Martin Stocker

**Affiliations:** a Department of Intensive Care and Neonatology, and Children's Research Center, University Children's Hospital Zürich, University of Zürich, Zurich, Switzerland; b Precision Medicine Unit, Biomedical Data Science Center, Lausanne University Hospital and University of Lausanne, Lausanne, Switzerland; c Global Health Institute, School of Life Sciences, École Polytechnique Fédérale de Lausanne, Lausanne, Switzerland; d Neonatal Intensive Care Unit, San Matteo Research Hospital, Pavia, Italy; e Neonatal Unit, Geneva University Hospitals, Mother, Child and Adolescent Department, Geneva, Switzerland; f Clinic of Neonatology, Department Woman-Mother-Child, Lausanne University Hospital and University of Lausanne, Lausanne, Switzerland; g Divisions of Allergy and Immunology, University Children's Hospital Zurich and Children's Research Center, University of Zürich, Zurich, Switzerland; h Pediatric Intensive Care and Neonatology, Children's Hospital of Central Switzerland and University of Lucerne, Lucerne, Switzerland; i Pediatric Infectious Diseases Unit, Children's Hospital of Geneva, University Hospitals of Geneva, Geneva, Switzerland; j Infectious Diseases and Vaccinology, University of Basel Children's Hospital, Basel, Switzerland; k Children's Hospital Aarau, Aarau, Switzerland; l Division of Infectious Diseases and Infection Control, Children's Hospital of Eastern Switzerland St. Gallen, St. Gallen, Switzerland; m Department of Neonatology, University Hospital Zürich, Zurich, Switzerland; n Division of Infectious Diseases and Hospital Epidemiology, and Children's Research Center, University Children's Hospital Zürich, Zurich, Switzerland; o Division of Infectious Disease, Department of Pediatrics, Inselspital, Bern University Hospital, University of Bern, Bern, Switzerland; p Child Health Research Centre, The University of Queensland, Brisbane, Australia

**Keywords:** Paediatric, Sepsis, Bacteraemia, Genome-wide association study, CTNNAL1, ELP1

## Abstract

**Background:**

Sepsis is defined as a dysregulated host response to infection leading to organ dysfunction. It represents a major global health concern, particularly in childhood. The underlying pathophysiological and genetic mechanisms remain insufficiently understood.

**Methods:**

Using samples and clinical data from 650 children enrolled in the Swiss Pediatric Sepsis Study, a national multicentre cohort for culture-proven bacterial sepsis, we conducted within-cohort analyses and a separate case–control analysis in 510 cases and 994 controls, testing genome-wide polymorphisms for association with sepsis susceptibility and, in cases only, with disease characteristics.

**Findings:**

In the within-cohort analysis, no significant genome-wide associations were found when assessing host, microbiological, and outcome features. In the case–control analysis, we identified one locus significantly associated with sepsis susceptibility, encompassing the *CTNNAL1* and *ELP1* genes.

**Interpretation:**

Our results suggest contribution of genetic modulators to susceptibility for sepsis in children.

**Funding:**

The Swiss Pediatric Sepsis Study received funding from the Swiss National Science Foundation (342730_153158/1 and 320030_201060/1), the Swiss Society of Intensive Care, the Bangerter Foundation, the Vinetum and Borer Foundation, the Foundation for the Health of Children and Adolescents, and the Sanofi-Aventis Suisse. LJS was supported by the NOMIS and the Thomas and Doris Ammann Foundation.

**Research in context**

**Evidence before this study**

Bacterial sepsis in children remains an important cause of morbidity and mortality. The Swiss Pediatric Sepsis Study has defined its incidence, clinical features, and outcomes in Switzerland. This nationwide cohort study spanning 2011 until 2015 reported an incidence of 25.1 per 100,000 children annually and a 30-day mortality rate of 7%, with risk of death closely associated with the presence and number of organ dysfunctions. Distinct clinical subgroups related to age and comorbidities were associated with specific pathogens, risk profiles, and outcomes. While many studies have reported on epidemiology of sepsis, studies investigating the susceptibility to sepsis in children are scarce. While rare mendelian diseases, in particular primary immunodeficiencies, may explain a minority of septic patients with extreme phenotypes, overall the contribution of genetic risk factors to sepsis in children remains poorly understood. Genome-wide association studies (GWAS) in adults with sepsis have yielded conflicting results. A GWAS on susceptibility to menincococcal infections revealed association with complement-related genes (Davila et al., 2010). To the best of our knowledge as of April 2026, there are no population-based studies using GWAS in children with blood culture-proven sepsis.

**Added value of this study**

This genome-wide association study focuses on paediatric bacterial sepsis based on a national cohort with blood culture-confirmed diagnoses. The cohort included 650 sepsis cases and 1395 population-based controls. Within-cohort analyses did not reveal significant association with host, microbiological, or outcome features. In case control analyses restricted to 510 cases and 994 controls of European ancestry, a genome-wide significant susceptibility locus was identified on chromosome 9 (*CTNNAL1*/*ELP1*). The study expands previous Swiss Pediatric Sepsis Study research by integrating epidemiological data with genome-wide genetics and by applying stringent ancestry correction and imputation within the clinical phenotype. The findings point towards new avenues for investigating genetic contributions to paediatric sepsis susceptibility.

**Implications of all the available evidence**

Applying GWAS to a well characterised population-based cohort with a microbiologically confirmed phenotype allowed us to investigate the contribution of both genetic and clinical factors to sepsis susceptibility and outcome in children. Pending independent validation in other cohorts, the identification of a susceptibility locus near *CTNNAL1* and *ELP1* provides a potential target for mechanistic studies and implicates transcriptional regulation and NF-κB signalling pathways. The use of a microbiologically confirmed phenotype in this cohort enhances interpretability and relevance for translational research. These results can inform future work on host–pathogen interactions, investigations of genetic risk, and justify further research on targeted interventions in paediatric sepsis.

## Introduction

Sepsis represents one of the leading causes of death and disability in adults and children.^1^^,^^2^^,^^3^^,^^4^^,^^5^ Sepsis has been defined as the result of a dysregulated host response to infection leading to organ dysfunction, however, the genetic and biomolecular mechanisms underpinning the susceptibility and pathway to ultimately detrimental host–pathogen interactions remain poorly understood.^6^^,^^7^^,^^8^ Sepsis in children is caused by a complex and heterogeneous pathogenesis, most commonly triggered by bacterial infections–accordingly, timely administration of intravenous antibiotics remain a key pillar of initial management recommendations.^9^

The Swiss Pediatric Sepsis Study is a national observational multicenter cohort study investigating blood culture-proven bacterial sepsis in children in Switzerland. Clinical and laboratory data have been have been previously reported.^4^^,^^10^^,^^11^ The age-standardised incidence of sepsis was 25.1 per 100,000 children per year with an average 30-day in-hospital mortality of 6.9% (95% CI 5.6–8.6), with highest rates observed in children under five years of age.

The incidence of sepsis is highest during early life,^12^ a vulnerable period of adaptive immunity development.^13^^,^^14^^,^^15^ While mechanisms underlying susceptibility and resistance to severe infectious diseases in otherwise healthy patients remains largely unknown, epidemiological studies suggest a genetic contribution.^16^^,^^17^ Previous studies have used mostly candidate gene and linkage analyses.^18^ Genome-wide association studies (GWAS), which allow a comprehensive interrogation of potential associations throughout the genome, have identified common genetic variants that are associated with many infectious diseases.^19^ Understanding why a minority of children develop invasive bacterial infections and become very ill remains one of the unsolved challenges in host genetic research, and may harbour clues for future targeted interventions. We here use GWAS applied to a national population-based cohort of children with blood culture-proven sepsis to elucidate host genetic determinants of susceptibility to paediatric sepsis and disease characteristics.

## Methods

### Ethics statement

The study was approved by the respective ethics committees of all participating centres (Cantonal Ethics Committee Bern, approval number KEK-029/11) and the study was conducted in accordance with the Declaration of Helsinki. Parents of participants provided their written consent to participate in the study after having been informed about the nature and purpose of the study, participation/termination conditions, and the risks and benefits of participation.

### Cohort description

The Swiss Pediatric Sepsis Study was a nationwide, prospective, multicentre cohort conducted from September 1, 2011, to December 31, 2015, across 10 tertiary and regional paediatric hospitals in Switzerland, including all five university centres and all paediatric intensive care units. Details of this cohort have been reported previously.^3^^,^^20^ Recruitment was consecutive and based on predefined eligibility criteria: children under 17 years of age with blood culture–proven bacterial or fungal sepsis, defined according to the 2005 International Paediatric Sepsis Consensus Conference (IPSCC) criteria requiring systemic inflammatory response syndrome at the time of blood culture sampling.^1^^,^^4^^,^^5^ Blood culture contaminants, post–allogeneic bone marrow transplant cases, and cases of asymptomatic bacteraemia not meeting systemic inflammatory response syndrome criteria were excluded.

During the study period, a total of 1269 blood culture-proven sepsis episodes were recorded in 1164 children. Participating centres accounted for 77.6% of all paediatric hospital admissions and 97.8% of all PICU admissions in Switzerland with an ICD-10 code for pathogen-specific sepsis. Clinical, laboratory, and outcome data were prospectively captured using standardised case report forms.

Episodes were classified as community- or hospital-acquired according to timing of culture. Comorbidities were coded using the validated Paediatric Complex Chronic Conditions classification system (v2). Organ dysfunction was adjudicated using the 2005 IPSCC definitions (IPSCC), PELOD-2, and pSOFA.^1^^,^^4^^,^^5^ Outcomes included 30-day mortality and length of hospital and PICU stay.

### Genotype quality control and imputation

Genomic DNA was extracted from whole blood of 704 participants. From these, 670 were patients with clinical features and genomic data suitable for our analysis. Samples were genotyped using Illumina OmniExpressExome-8 v1.4 genotyping array and genotypes were called using Illumina GenomeStudio. An in-house control cohort of 1395 individuals from a Swiss population-based GWAS was included. Controls were also genotyped on Illumina OmniExpress arrays and processed under the same quality control and imputation pipeline as cases. Variant-level filters were applied consistently across both groups, and population structure was aligned using principal component analysis (PCA) to remove outliers. As the analyses targeted common germline variants, this population-based cohort served as a suitable reference group for case–control comparison.

Study participants were excluded based on a missing genotype call rate of 10%. Subject independence was assessed using KING; nine samples were removed due to a high degree of kinship or duplication (pairwise identify-by-state (IBS) estimated kinship coefficient >0.18).^21^ From the distribution of genetic distance within cases, no exclusion of outliers was necessary. One sample was removed based on retraction of genetic consent.

Variants were removed for minor allele frequencies <0.05, missingness >0.1, and additionally for controls, Hardy–Weinberg Equilibrium (HWE) P < 1E-6. Reported and estimated sex was examined for discrepancy. We compared the genetic ancestry in cases to self-reported ethnicity to check for mislabelling. After quality control (QC), 650 sepsis case samples and 1395 controls remained. Genotyping data was phased (SHAPEIT2) and imputed (IMPUTE2) using the 1000 Genomes Project phase 3 reference panel.^22^^,^^23^ The reference genome build and LD population used was hg19/1000G Nov2014 EUR. Imputation quality was assessed and SNPs with an information score of <0.8 or minor allele frequency <0.05 were removed (Figure S2).

Because uneven genotype coverage and imputation quality can generate isolated extreme SNP association signals without surrounding support, an additional variant-level QC step was applied to reduce likely technical artefacts. Support was assessed within a 250 kb window and anchored by the presence of at least one high-confidence imputed SNP (INFO ≥0.99). The support threshold was selected empirically to reduce obvious singleton outlier peaks while preserving broader association structure. Analysis with and without this QC are provided in the data repository.

### Association testing

The Genome-wide Complex Trait Analysis (GCTA) software package was used to calculate the genetic relationship matrix (GRM) and PCA to quantify population structure.^24^ Within-cohort (case only) association testing was performed on all 650 case samples. For case–control analysis of sepsis susceptibility, population structure/stratification was alleviated by sub-setting the dataset to remove outlying clusters as evident from PCA (Figure S3). This removed 140 cases and 401 controls, leaving a case–control subset of 510 cases and 994 controls of European ancestry. Datasets were merged using PLINK v1.9. SNP positions and identifiers were updated according to dbNSFP4.0a (hg19).^25^ QC was repeated after merging cases and controls for combined cohort-specific frequencies and GCTA mixed linear model-based association (MLMA) with leaving-one-chromosome-out (LOCO) was used for analysis. SNP QC filters and association model summaries are listed in Tables S1 and S2, respectively. GCTA MLMA-LOCO was used for within-cohort (case only) analyses of several phenotypes as listed in Table 1 and Table S2.^26^ Population structure was controlled by GRM eigenvectors and analysis covariates consisted of sex, age, and study site.

**Table 1:** Demographic and clinical characteristics of 650 Swiss Pediatric Sepsis Study GWAS participants.

	All children	Previously healthy children	Neonates	Children with comorbidities
	(n = 650)	(n = 216)	(n = 220)	(n = 214)
Age at sepsis onset (months)	9.4 (0.6–69.7)	51.8 (11.0–121.4)	0.3 (0.1–0.6)	35.5 (8.4–95.3)
Age groups				
Preterm newborn	125 (19%)		125 (57%)	
Term newborn (<28 days)	95 (15%)		95 (43%)	
28–365 days	124 (19%)	59 (27%)		65 (30%)
1–4 years	129 (20%)	57 (26%)		72 (34%)
5–9 years	85 (13%)	44 (20%)		41 (19%)
10–16 years	92 (14%)	56 (26%)		36 (17%)
Sex				
Female sex	254 (39%)	79 (37%)	86 (39%)	89 (42%)
Male sex	396 (61%)	137 (63%)	134 (61%)	125 (58%)
Ethnic origin^a^				
White European	541 (83%)	188 (87%)	189 (86%)	164 (77%)
Asian	20 (3%)	8 (4%)	4 (2%)	8 (4%)
African	30 (5%)	6 (3%)	3 (1%)	21 (10%)
Arabian	2 (<1%)		1 (<1%)	1 (<1%)
Jewish	1 (<1%)		1 (<1%)	
Other Mid eastern	3 (<1%)		3 (1%)	
Mixed ethnicity	34 (5%)	6 (3%)	15 (7%)	13 (6%)
Comorbidities^b^				
Neurological or neuromuscular	9 (1%)		2 (1%)	7 (3%)
Cardiovascular	21 (3%)		2 (1%)	19 (9%)
Respiratory	1 (<1%)			1 (<1%)
Renal and urological	19 (3%)		2 (1%)	17 (8%)
Gastrointestinal	13 (2%)		1 (<1%)	12 (6%)
Haematological or immunological	6 (1%)			6 (3%)
Metabolic	2 (<1%)			2 (1%)
Other congenital or genetic defect	1 (<1%)			1 (<1%)
Malignant disease	69 (11%)			69 (32%)
Neonatal	58 (9%)		58 (26%)	
Surgery or burn	21 (3%)		4 (2%)	17 (8%)
Technology dependence	18 (3%)		14 (6%)	4 (2%)
More than one condition	97 (15%)		39 (18%)	58 (27%)
Hospital-acquired sepsis?				
Early-onset sepsis	53 (8%)		53 (24%)	
Community-acquired sepsis	372 (57%)	205 (95%)	51 (23%)	116 (54%)
Hospital-acquired sepsis	225 (35%)	11 (5%)	116 (53%)	98 (46%)
CVAD present at time of sepsis onset				
No	427 (66%)	216 (100%)	137 (62%)	74 (35%)
Yes	223 (34%)		83 (38%)	140 (65%)
Length of stay after sepsis onset (days)	15 (10–31)	10 (7–16)	22 (14–64)	17 (10–35)
Adequate empirical antibiotic treatment^c^				
Yes	562 (86%)	199 (92%)	189 (86%)	174 (81%)
No	87 (13%)	17 (8%)	30 (14%)	40 (19%)
Length of antibiotic treatment (days)^d^	14 (10–16)	14 (10–26)	14 (10–14)	14 (12–17)
Site or type of infection				
Primary bloodstream	119 (18%)	21 (10%)	74 (34%)	24 (11%)
Central line-associated bloodstream	162 (25%)		68 (31%)	94 (44%)
Urinary tract	72 (11%)	25 (12%)	27 (12%)	20 (9%)
Pneumonia	64 (10%)	46 (21%)	10 (5%)	8 (4%)
Central nervous system	53 (8%)	33 (15%)	15 (7%)	5 (2%)
Gastrointestinal system	40 (6%)	13 (6%)	7 (3%)	20 (9%)
Bones and joints	48 (7%)	40 (19%)	4 (2%)	4 (2%)
Skin and soft tissue	30 (5%)	15 (7%)	6 (3%)	9 (4%)
Ear, nose, and throat	17 (3%)	12 (6%)	3 (1%)	2 (1%)
Surgical site	13 (2%)		4 (2%)	9 (4%)
Cardiovascular system	11 (2%)		1 (<1%)	10 (5%)
Toxic shock syndrome	6 (1%)	5 (2%)		1 (<1%)
Other specific infection type	15 (2%)	6 (3%)	1 (<1%)	8 (4%)
Pathogens identified^e^				
*Staphylococcus aureus*	94 (14%)	42 (19%)	17 (8%)	35 (16%)
Coagulase-negative staphylococci	79 (12%)		56 (25%)	23 (11%)
*Streptococcus pneumoniae*	64 (10%)	51 (24%)	2 (1%)	11 (5%)
Viridans group streptococci	30 (5%)	6 (3%)	5 (2%)	19 (9%)
Group A streptococci	36 (6%)	33 (15%)	1 (<1%)	2 (1%)
Group B streptococci	54 (8%)	13 (6%)	41 (19%)	
*Enterococcus spp*	16 (2%)	2 (1%)	8 (4%)	6 (3%)
Other Gram-positive bacteria	16 (2%)	5 (2%)	8 (4%)	3 (1%)
*Escherichia coli*	116 (18%)	24 (11%)	49 (22%)	43 (20%)
*Haemophilus influenzae*	10 (2%)	9 (4%)	1 (<1%)	
*Klebsiella spp*	28 (4%)		10 (5%)	18 (8%)
*Neisseria meningitidis*	17 (3%)	16 (7%)		1 (<1%)
*Pseudomonas aeruginosa*	16 (2%)	1 (<1%)	2 (1%)	13 (6%)
Other Gram-negative bacteria	53 (8%)	13 (6%)	11 (5%)	29 (14%)
*Candida albicans*	12 (2%)		3 (1%)	9 (4%)
Number of organ dysfunctions^f^				
None	281 (43%)	135 (62%)	60 (27%)	86 (40%)
One	168 (26%)	29 (13%)	83 (38%)	56 (26%)
Two	78 (12%)	15 (7%)	40 (18%)	23 (11%)
Three	62 (10%)	16 (7%)	24 (11%)	22 (10%)
Four	31 (5%)	6 (3%)	8 (4%)	17 (8%)
Five	22 (3%)	12 (6%)	3 (1%)	7 (3%)
Six	5 (1%)	2 (1%)	1 (<1%)	2 (1%)
PICU or NICU admission				
No	327 (50%)	142 (66%)	55 (25%)	130 (61%)
Yes	323 (50%)	74 (34%)	165 (75%)	84 (39%)
PICU or NICU admission due to sepsis				
No	10 (2%)	2 (1%)	6 (3%)	2 (1%)
Yes	171 (26%)	70 (32%)	64 (29%)	37 (17%)
Length of PICU stay (days)	14 (4–52)	4 (2–9)	24 (6–67)	15 (3–70)
Length of PICU stay due to sepsis (days)	11 (3–35)	4 (2–9)	17 (5–54)	10 (3–35)
Invasive ventilation				
No	476 (73%)	176 (81%)	130 (59%)	170 (79%)
Yes	174 (27%)	40 (19%)	90 (41%)	44 (21%)
Invasive ventilation due to sepsis				
No	51 (8%)	2 (1%)	29 (13%)	20 (9%)
Yes	123 (19%)	38 (18%)	61 (28%)	24 (11%)
Non-invasive ventilation				
No	546 (84%)	206 (95%)	134 (61%)	206 (96%)
Yes	104 (16%)	10 (5%)	86 (39%)	8 (4%)
Non-invasive ventilation due to sepsis				
No	58 (9%)	1 (<1%)	51 (23%)	6 (3%)
Yes	46 (7%)	9 (4%)	35 (16%)	2 (1%)
Inotrope requirement				
No	531 (82%)	178 (82%)	182 (83%)	171 (80%)
Yes	119 (18%)	38 (18%)	38 (17%)	43 (20%)
Case fatality				
Survived	629 (97%)	213 (99%)	209 (95%)	207 (97%)
Died	21 (3%)	3 (1%)	11 (5%)	7 (3%)
Time to death from sepsis onset (days)	8 (3–24)	2 (0–5)	5 (3–20)	16 (9–73)

Categorical variables are presented as frequencies (%) and continuous variables as median (IQR). Column percentages are presented based on available data for each variable.

CVAD, central venous access device; PICU, paediatric intensive care unit; NICU, neonatal intensive care unit.

a Missing in 19.

b Missing in 1.

c Missing in 1.

d Missing in 3.

e Missing in 9.

f In 3 cases laboratory and physiological parameters needed to define organ dysfunctions according to the 2005 consensus definition were missing, but according to the initial investigator-based classification these patients did not suffer from any organ dysfunction.

### Regional association and LD analysis

Regional association was assessed using summary statistics centred on the lead variant at the FAM206A/CTNNAL1 locus (chr9:111.6–111.7 Mb). Reference linkage disequilibrium (LD) was obtained via *LDlinkR* using 1000 Genomes Phase 3 populations.^27^^,^^28^ Pairwise r^2^ values to the lead SNP were extracted for all genetic ancestry panels and merged with GWAS z-scores to assess concordance between reference and cohort LD. Fine-mapping was performed with *susieR* (‘*susie_rss’*) using z-scores and the reference LD matrix to infer 95% credible sets and posterior inclusion probabilities (PIP). Conditional association diagnostics were derived by comparing observed z-score ratios (zᵢ/z_lead) to reference correlations (r) and by computing conditional z-scores (‘*z_cond* = *z − r* × *z_lead*’) to test for residual association after conditioning on the lead variant. Cross-population LD profiles were calculated using *LDlinkR* and visualised to compare r^2^ decay across 1000 Genomes populations, highlighting population-specific differences in LD structure that influence locus appearance.

### SuSiE and GTEx component

Fine mapping was conducted with SuSiE using summary statistics from the case control analysis and reference linkage disequilibrium from 1000 Genomes EUR obtained with LDlinkR.^27^^,^^28^ Z scores were calculated from the effect estimates and standard errors. SuSiE was run with three effect components, 95 per cent coverage, and a reference correlation matrix corrected to the nearest positive definite matrix.^29^^,^^30^ Credible sets and posterior inclusion probabilities were extracted from the fitted model. Expression and splicing QTL associations for rs28361152 were taken from GTEx v8.^31^ Significant variant gene tissue pairs were extracted from the eQTL and sQTL tables and normalised effect sizes were used to summarise direction and magnitude of regulatory activity across tissues. Expression and splicing QTLs were obtained from the GTEx v8 significant QTL release, which applies a Benjamini-Hochberg false discovery rate threshold of 0.05 to SNP-gene pairs within a ±1 Mb cis window in each tissue. Reported P values correspond to the nominal GTEx statistics.^31^

### Sex/gender reporting

Genetic sex was assessed based on genotype data and compared with data self-reported by study participants to check for mismatch. Genetic sex was used as a covariate during analysis.

### Role of funders

The funders were not involved in study design, data collection and analysis, interpretation, and writing of the manuscript.

## Results

The demographic and clinical characteristics of the 650 study participants for whom within-cohort analysis was performed are shown in Table 1 and Figure S1. Within-cohort (case only) association testing was performed on all 650 case samples. We found no significant association of genetic variants with specific sepsis characteristics, including site or type of infection, cardiovascular failure, comorbidities, pathogens identified, case fatality, PICU admission, hospital-acquired sepsis, length of PICU stay, time to death from sepsis onset, number of organ dysfunctions, length of stay after sepsis onset, invasive ventilation, and age groups (Table S2, Figure S7).

**Fig. 1:**
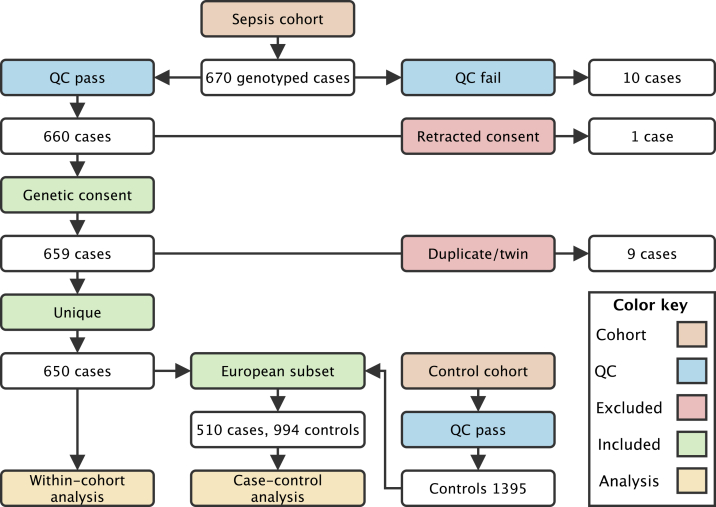
**Cohort analysis summary.** Swiss Pediatric Sepsis cohort episodes, genotyping, and quality control. Within-cohort analysis are listed in Table S2. Case-control analysis subset to suitably match cohorts.

**Fig. 2:**
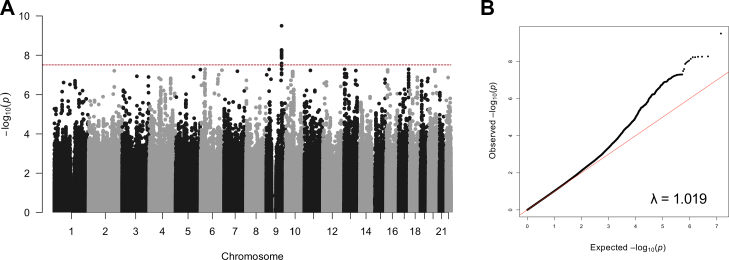
**GWAS Manhattan plot and QQ plot.** (A) Manhattan plot of association shows significant association signals in chromosome 9 (significant threshold P-value = 5e^−08^). (B) QQ plot demonstrates that the observed distribution of P-values corresponds to the expected distribution under the null hypothesis, indicating that potential confounders are well controlled, λ = 1.019.

A subset of 510 cases and 994 Swiss population-matched controls were included in a case–control GWAS for sepsis susceptibility as shown in Fig. 1. This analysis highlighted one significantly associated region of chromosome 9 with 13 genome-wide significant SNPs and rs28361152 as the top-associated variant (Table 2 and Fig. 2A). Conditional analysis with GCTA-COJO showed that all SNPs reflect the same association signal. All 13 SNPs were non-coding and did not initially appear to affect protein structure, but deeper analyses revealed their functional effects as expression Quantitative Trait Loci (eQTL). The QQ-plot in Fig. 2B shows no systematic genomic inflation, supporting the validity of the association results shown in Fig. 2A.

**Table 2:** Significant associations identified for case and control susceptibility.

Chr	refSNP ID	Pos	A1	A2	Freq	beta	SE	P
9	rs11788336	111688387	C	T	0.230	−0.11	0.02	5.34E-09
9	rs113624314	111689953	A	G	0.229	−0.11	0.02	5.88E-09
9	rs744980	111690388	T	C	0.229	−0.11	0.02	5.88E-09
9	rs3763643	111695607	C	T	0.229	−0.11	0.02	7.25E-09
9	rs2275637	111696043	G	C	0.229	−0.11	0.02	1.02E-08
9	rs7027263	111699569	A	G	0.230	−0.11	0.02	5.50E-09
9	rs28361186	111704064	A	G	0.231	−0.11	0.02	1.16E-08
9	rs12683697	111709659	T	C	0.233	−0.10	0.02	2.57E-08
9	rs7028937	111711861	T	C	0.231	−0.11	0.02	1.36E-08
9	rs11791735	111713520	T	C	0.231	−0.11	0.02	8.97E-09
9	rs28361152	111727529	C	T	0.227	−0.12	0.02	3.12E-10
9	rs74384717	111729664	TCA	T	0.232	−0.11	0.02	5.57E-09
9	rs11790105	111731785	C	T	0.233	−0.10	0.02	3.28E-08

Lead SNP rs28361152 is a non-coding variant that falls within *CTNNAL1*. The remaining associated SNPs in LD (r^2^ ≥ 0.8) span *ELP1* and *FAM206A*.

**Fig. 3:**
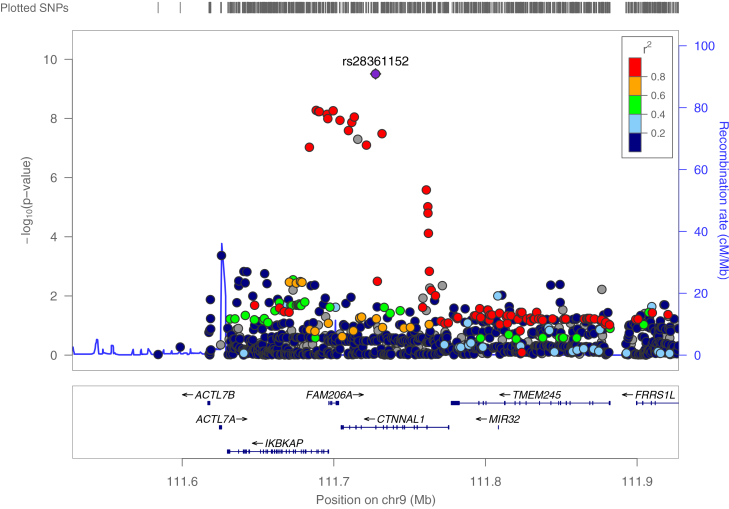
**A LocusZoom plot for SNPs in the region flanking 200 kb on either side of the selected SNP rs28361152 on chromosome 9.** P-values in −log10 scale are shown on the left vertical axis, the recombination rates are on the right vertical axis, and the chromosomal positions are on the horizontal axis. LD is shown by r^2^ and represented by colour. The lead SNP is in strong LD with nearest gene *CTNNAL1*, *FAM206A,* and *ELP1.* The LD block extends to *TMEM245* and *FRRS1L,* but no associated with disease is evident within the Swiss Pediatric Sepsis Study cohort.

The lead SNP, associated variants and gene positions in this region are shown in a LocusZoom plot in Fig. 3. The strength and extent of the association signal relative to genomic position, local LD and recombination patterns indicate five genes of potential interest: *ELP1* (elongator acetyltransferase complex subunit 1), *FAM206A* (family with sequence similarity 206, member A), *CTNNAL1* (catenin alpha-like 1 gene), *TMEM245* (Transmembrane protein 245), and *FRRS1L* (DOMON domain-containing protein FRRS1L). Using GTEx v.8 data (Figure S4), we observed that the top associated variant is a significant cis-eQTL for all five genes in the LD block (Fig. 3). Regional LD diagnostics supported one association signal at this locus rather than multiple independent signals (Figure S5).

The regional association peak at 111.6–111.7 Mb identified a single lead variant (P ≈ 10^−10^). When coloured by reference LD using LocusZoom, variants extending into the neighbouring 111.7–111.8 Mb region appear red, reflecting high r^2^ in the 1000G EUR panel, yet these variants do not show low P values in the GWAS cohort. The z versus r diagnostic confirms that correlations to the lead are weaker in our data than in the reference panel. After conditioning on the lead using reference LD, the residual association largely disappears, indicating that the signal is consistent with one independent effect. The modest residual in the right-hand region is most consistent with LD mismatch between the reference and the cohort, including population-specific haplotype structure and incomplete transfer of tagging, rather than a second signal. Cross-population r^2^ profiles further show substantial variation in LD structure, with an extended block in European panels but rapid decay in African panels. Together, these analyses support a single association centred on the FAM206A locus, and the apparent extension in reference-based LD reflects population-specific haplotype differences rather than multiple signals. Cross-population r^2^ profiles further show that LD structure varies across populations.

Fine mapping and regulatory analyses were undertaken to characterise the functional context of the putative susceptibility locus. All coding and non-coding variants in LD with the lead SNP are reported in Tables S3 and S4. Table S5 lists significant eQTLs for the 13 variants associated with susceptibility, with rs28361152 showing the strongest expression effect for *FRRS1L*. rs28361152 also acts as an sQTL for *ELP1* (Table S6). Genes in the region are widely expressed across tissues, apart from *FRRS1L* which shows overall low baseline expression (Figure S4). SuSiE fine mapping identified a single credible set, with rs28361152 receiving a posterior inclusion probability of 1.0 and all other variants approaching zero, consistent with one association signal (Figure S6A). GTEx v8 data showed that rs28361152 exerts regulatory effects across multiple tissues, including eQTL effects for *FRRS1L*, *TMEM245*, and *IKBKAP*, and sQTL effects restricted to *IKBKAP* (Figure S6B). These tissue dependent regulatory patterns are concordant with the genomic location of the association peak and support transcriptional regulation as a plausible mechanism linking the locus to sepsis susceptibility. The same locus maps to *ELP1* and *CTNNAL1* in an independent murine susceptibility screen of 73 Collaborative Cross lines challenged with *K. pneumoniae*, with joint discovery across studies highly unlikely by chance (P = 1.28 × 10^−13^, weighted Z test; P = 1.53 × 10^−17^, Fisher's method).^32^^,^^33^ Effect estimates for rs28361152 were directionally consistent in sepsis cases with and without *Klebsiella* infection, supporting a pathogen-independent association.

## Discussion

In this GWAS of a population-based national cohort study of children with blood culture proven sepsis, we explored associations with culture-proven bacteremia and sepsis-related outcomes for 650 cases and performed case–control analysis for 510 cases and 994 controls. Our search for human genetic determinants of susceptibility to paediatric sepsis did not identify any association meeting the statistical thresholds in the within-cohort analyses focussing on sepsis severity and pathogen features. The case–control study restricted to individuals of European ancestry identified a locus of interest on chromosome 9 with 13 genome-wide significant SNPs. The top associated variant, rs28361152, was an eQTL for five genes in the immediate vicinity - *ELP1, FAM206A*, *CTNNAL1*, TMEM245, and *FRRS1L*. It was also an sQTL for *ELP1*. Pending independent validation in other cohorts, these findings need to be considered preliminary. Of note, *elp1* and *ctnnal1* have been previously reported as candidate genes in a murine model of sepsis.^32^

*CTNNAL1* encodes alpha-catulin, a regulatory protein that is associated with inflammatory and immune pathways through modulation of the NF-kB pathway activity.^34^ Murine Ctnnal1 has been reported for its role in cell adhesion in response to bacterial triggers.^35^^,^^36^ *ELP1* encodes the elongator complex protein 1, a component of the RNA polymerase II elongator complex, which is involved in transcriptional elongation. This complex catalyses the formation of carboxymethyluridine in the wobble base at position 34 in tRNAs.^37^ Loss of mouse *Elp1* is embryonic lethal and can be rescued by human *ELP1*.^38^

Genome wide studies in adults have focused mainly on sepsis mortality, with heterogeneous and often non replicating results. Reported loci include *VPS13A, CRISPLD2*, and 13q21.33,^39^ regulatory variants at *CISH* and *MAPKAPK3*,^40^ and a missense variant in *SAMD9*.^41^ Integrative analyses combining sepsis genome-wide association data with Mendelian randomisation and transcriptomic resources have implicated additional pathways, including IL6R-mediated inflammatory signalling,^42^ metabolite-related susceptibility,^43^ and regulatory influences identified through transcriptome-wide association methods.^44^ A paediatric GWAS of meningococcal disease by Davila et al. identified and independently replicated strong associations at complement regulatory genes *CFH* and *CFHR3*, implicating host variation in complement activation as a determinant of invasive disease versus asymptomatic colonisation by *Neisseria meningitidis*.^45^ Paediatric GWAS-based data remain sparse. Transcriptomic studies of children with septic shock have demonstrated widespread repression of genes involved in adaptive immune function, including T cell receptor signalling, B cell function, and antigen presentation pathways, together with marked developmental age-dependent variation in host responses, as synthesised in Wong's review of a decade of genome-wide and transcriptomic investigations that also encompassed earlier discovery-era genetic studies,^46^ while a systematic review demonstrated substantial cross cohort heterogeneity.^47^ We previously reported on rare variants in known primary immunodeficiency genes, however many of these were of unknown significance.^20^

Susceptibility to sepsis may reflect the combination of susceptibility to severe bacterial infection, as well as susceptibility to dysregulated host response leading to greater severity. The Swiss Pediatric Sepsis Cohort was conducted in a high income country with rapid geographical access to paediatric hospitals and mandatory healthcare for all population. While the cohort was population-based, Swiss-specific characteristics related to patient comorbidities or age may not be representative of other settings. In the within-cohort study, we failed to identify any significant association of genetic variation with type or severity of infection. However, the study may have been underpowered for such analyses. Due to the cohort size, and lack of independent validation cohort, findings of the case–control study need to be considered preliminary. The *CTNNAL1* and *ELP1* locus has not been reported in adult survival or susceptibility studies. The association is supported indirectly by fine mapping, eQTL and sQTL integration, and concordance with Collaborative Cross mouse data.^32^

Despite the strengths of the Swiss Pediatric Sepsis Study including its comprehensive, longitudinal, population-based design and restriction to the microbiological goldstandard of invasive bacteria infection, several limitations need to be considered. The relatively small sample size limited the power of analyses. In particular, the case-only analyses were hindered by insufficient sample numbers, and the case–control analysis was constrained by the limited availability of well-matched controls for non-European genetic backgrounds. Differences in allele frequencies further highlighted the challenges in this research area. The in-house Swiss population control cohort revealed a minor allele frequency of approximately 0.3 for the lead variant, consistent with data from gnomAD and FinnGen. In contrast, African and South Asian populations exhibited much lower frequencies, 0.05 and 0.0, respectively. Increasing sample sizes and increasing the controls in future studies could enhance the ability to confirm and detect additional genetic associations more effectively. Genotype coverage was constrained by the array platform, and additional QC was needed to remove isolated signals likely to be false positives. In a study of this size, false positives are more likely than in larger GWAS. The chromosome 9 association should therefore be considered preliminary and requires independent replication before supporting biological or functional conclusions.

Although GWAS provided valuable statistical associations, they fall short of pinpointing causal variants, especially in non-coding regions where genomic complexity is a major hurdle. Replication studies with independent cohorts, along with functional assays and fine mapping, are needed to validate these associations and to provide a definitive explanation of the underlying mechanisms. Large-scale genomic approaches, such as targeted exome and whole genome sequencing (WGS), offer promising avenues for uncovering candidate genes and clarifying the biological mechanisms of host susceptibility and host–pathogen interactions. These insights may ultimately pave the way for targeted interventions. Currently, genomic investigations are not routinely performed in children with sepsis, despite the rapid uptake of WES and WGS in critically ill neonates and children.^9^^,^^48^

## Contributors

The Swiss Pediatric Sepsis Study Group (see Acknowledgements, Supplementary Material) contributed to study implementation and data collection. Individual members are listed in the manuscript according to their specific contributions. Dylan Lawless performed the formal analysis, wrote the manuscript, and accessed and verified the underlying data. Christian W. Thorball contributed to data collection, manuscript drafting, reproducibility study, and accessed and verified the underlying data. Flavia Aurelia Hodel, Zhi Ming Xu, Alessandro Borghesi, Eric Giannoni, Johannes Trück, Martin Stocker, Klara M Posfay-Barbe, Ulrich Heininger, Sara Bernhard-Stirnemann, Anita Niederer-Loher, Christian R. Kahlert, Giancarlo Natalucci, Christa Relly, Christoph Berger, Thomas Riedel, and Christoph Aebi contributed to data collection and manuscript drafting. Philipp Agyeman contributed to manuscript drafting, data curation, formal analysis, and accessed and verified the underlying data. Jacques Fellay and Luregn J. Schlapbach conceptualised the project, obtained funds, supervised the study, and contributed to writing the manuscript.

## Data sharing statement

Summary statistic data is available from the public repository in Zenodo https://doi.org/10.5281/zenodo.15675718. The summary statistics for this analysis are available in the GWAS Catalogue under accession GCST90726424. Access to individual-level genetic data is restricted due to research use agreements. Researchers may contact the corresponding authors (L.J.S. and J.F.) to request access, subject to institutional and ethical approvals.

Weblinks:

Git repository https://github.com/DylanLawless/spss_gwas.

Zenodo https://doi.org/10.5281/zenodo.15675718.

FinnGen https://r4.finngen.fi.

FUMA https://fuma.ctglab.nl.

GCTA https://cnsgenomics.com/software/gcta/

GTEx https://www.gtexportal.org/home/

HaploReg https://pubs.broadinstitute.org/mammals/haploreg/haploreg.php.

IMPUTE2 https://mathgen.stats.ox.ac.uk/impute/impute_v2.html.

KING https://people.virginia.edu/∼wc9c/KING/

LDLink https://ldlink.nci.nih.gov.

LocusZoom http://locuszoom.org.

PLINK http://zzz.bwh.harvard.edu/plink/

SHAPEIT2 https://mathgen.stats.ox.ac.uk/genetics_software/shapeit/shapeit.html.

## Declaration of interests

The authors declare no conflict of interest. Relevant funding for members of the Swiss Pediatric Sepsis Study is reported in the Funding section.

## Appendix A Supplementary data

Supplementary Tables

Supplementary Captions

Collab Authors
