# Supplementary figures and images for: Genome-wide association study of paediatric bacteraemia and sepsis
Source: eBioMedicine. 2026 Jun 4;129:106320. doi: 10.1016/j.ebiom.2026.106320 (PMC13266212; doi:10.1016/j.ebiom.2026.106320)

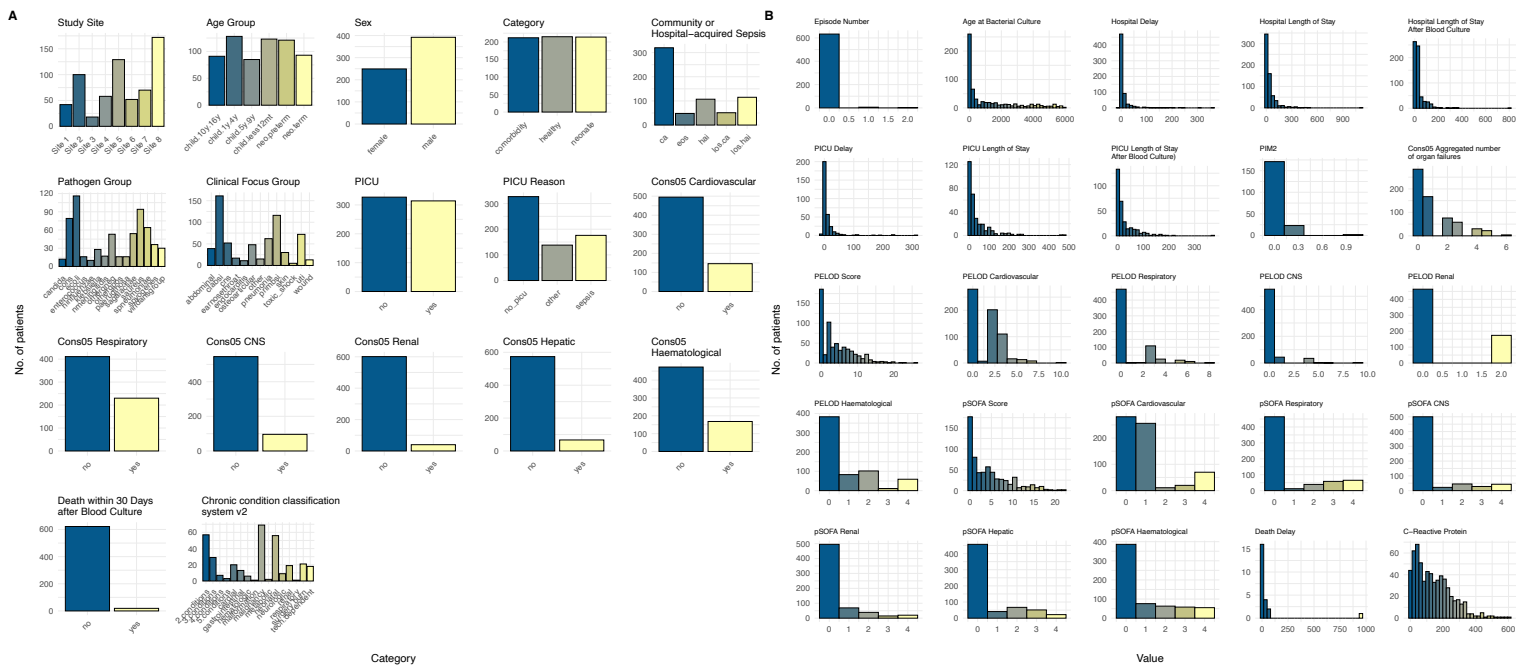

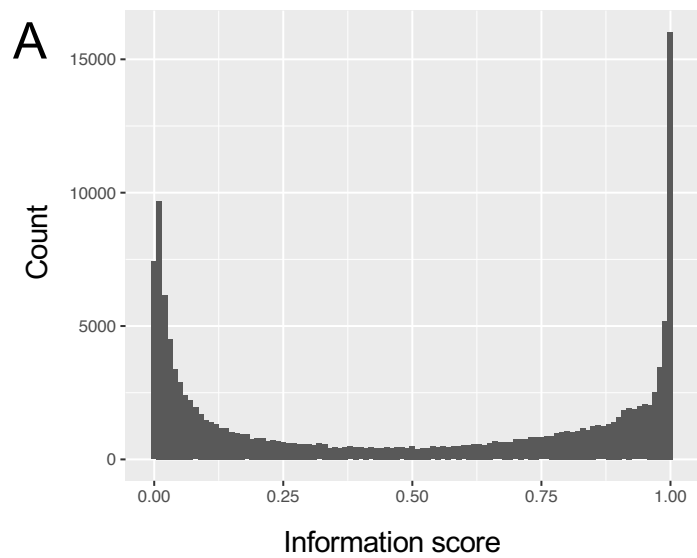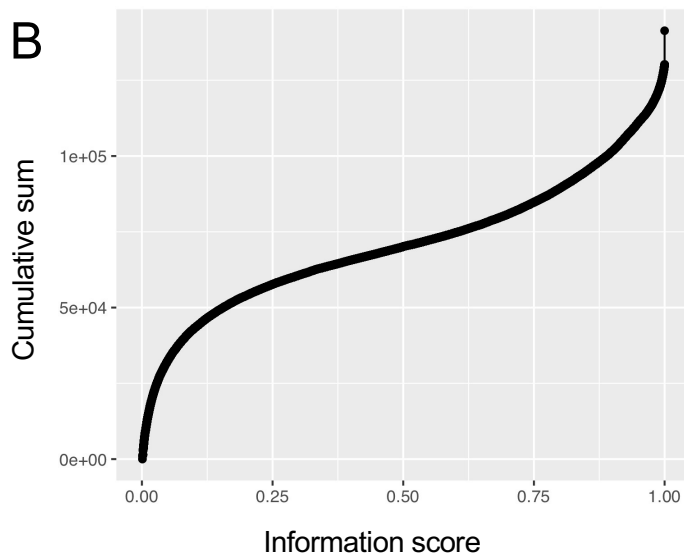

Figure S2

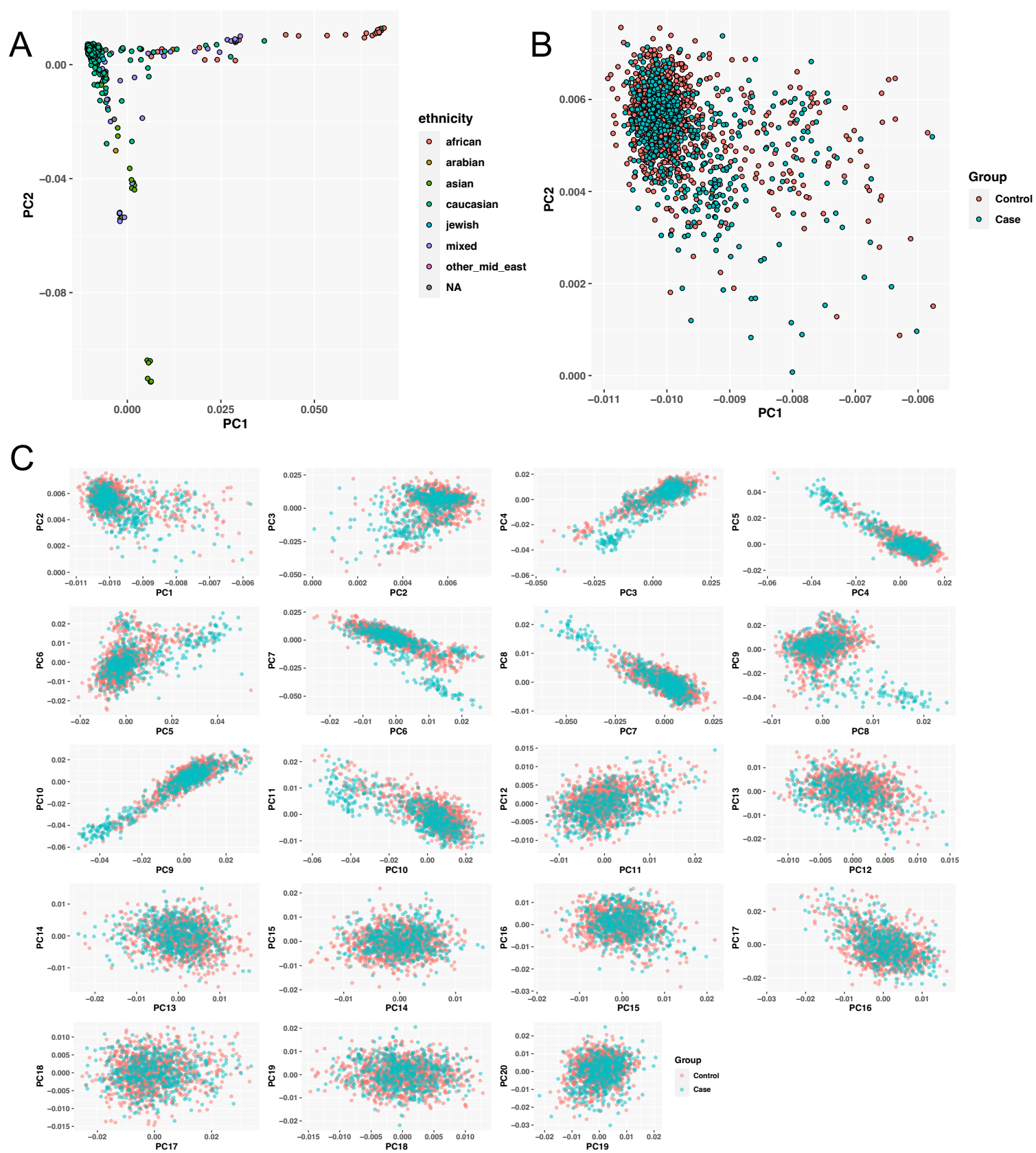

Figure S3

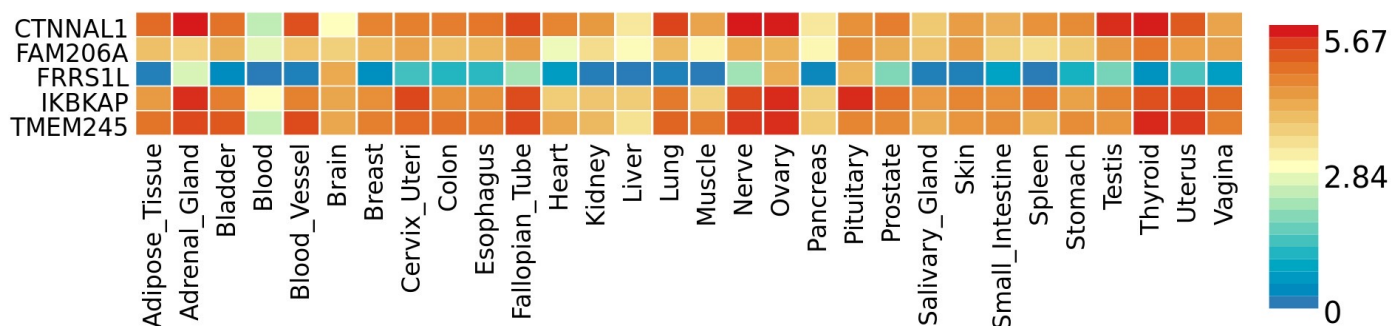

Figure S4

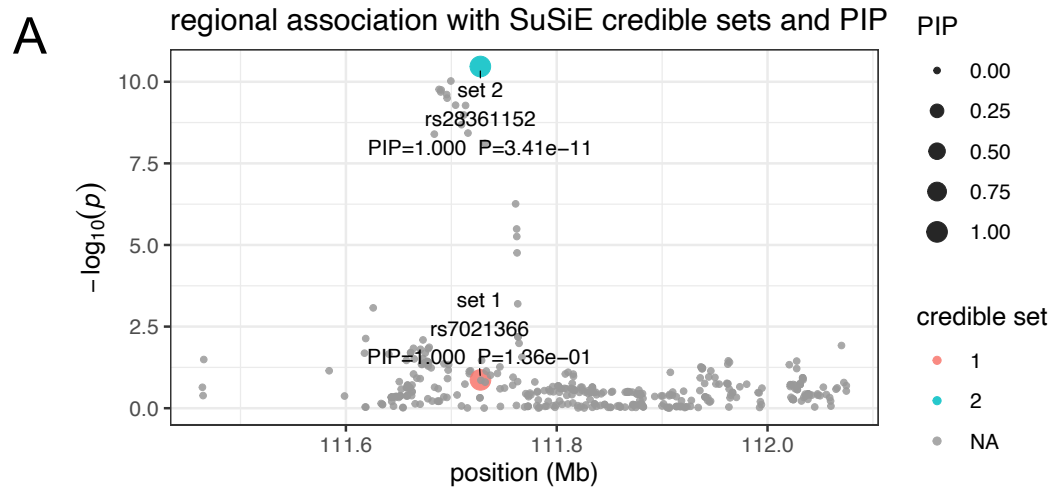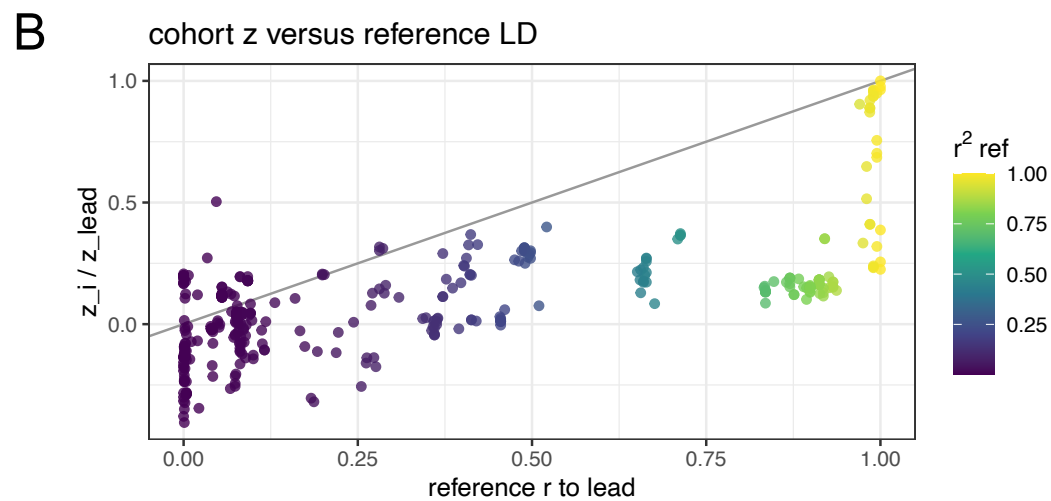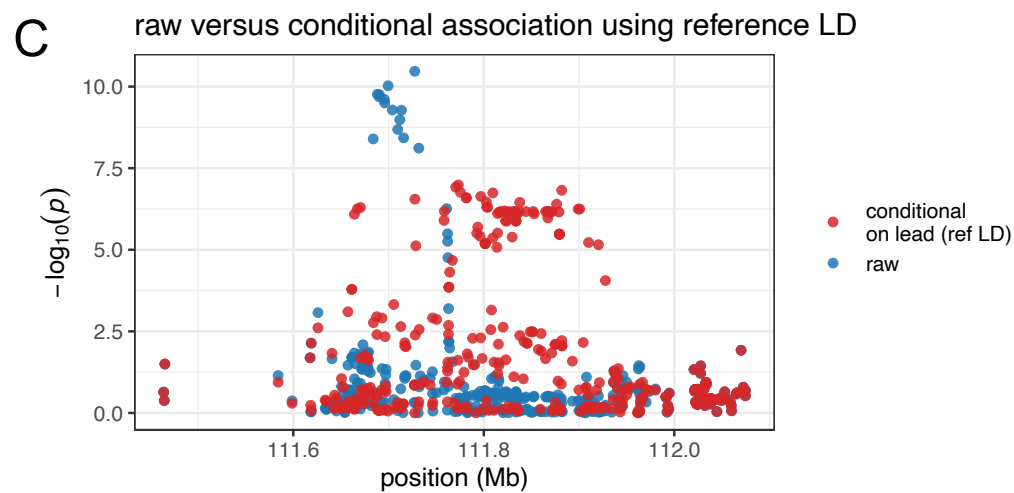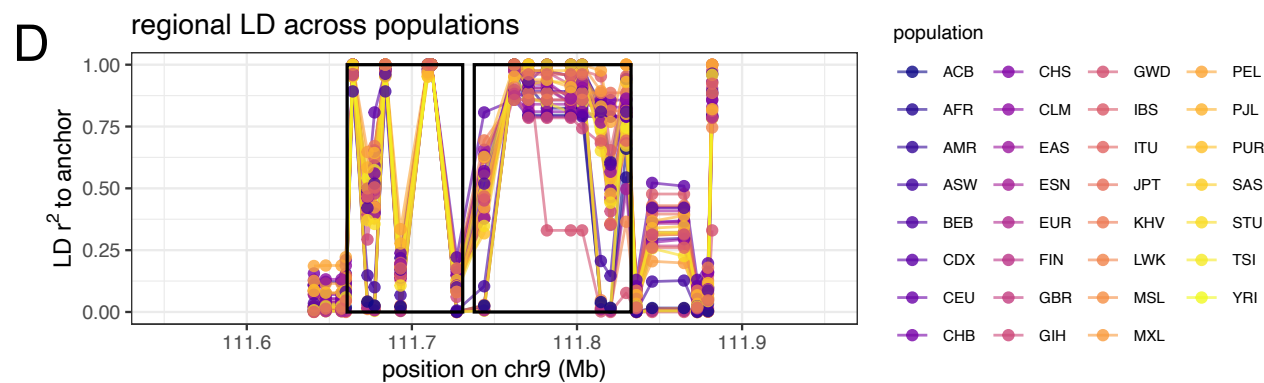

Figure S5

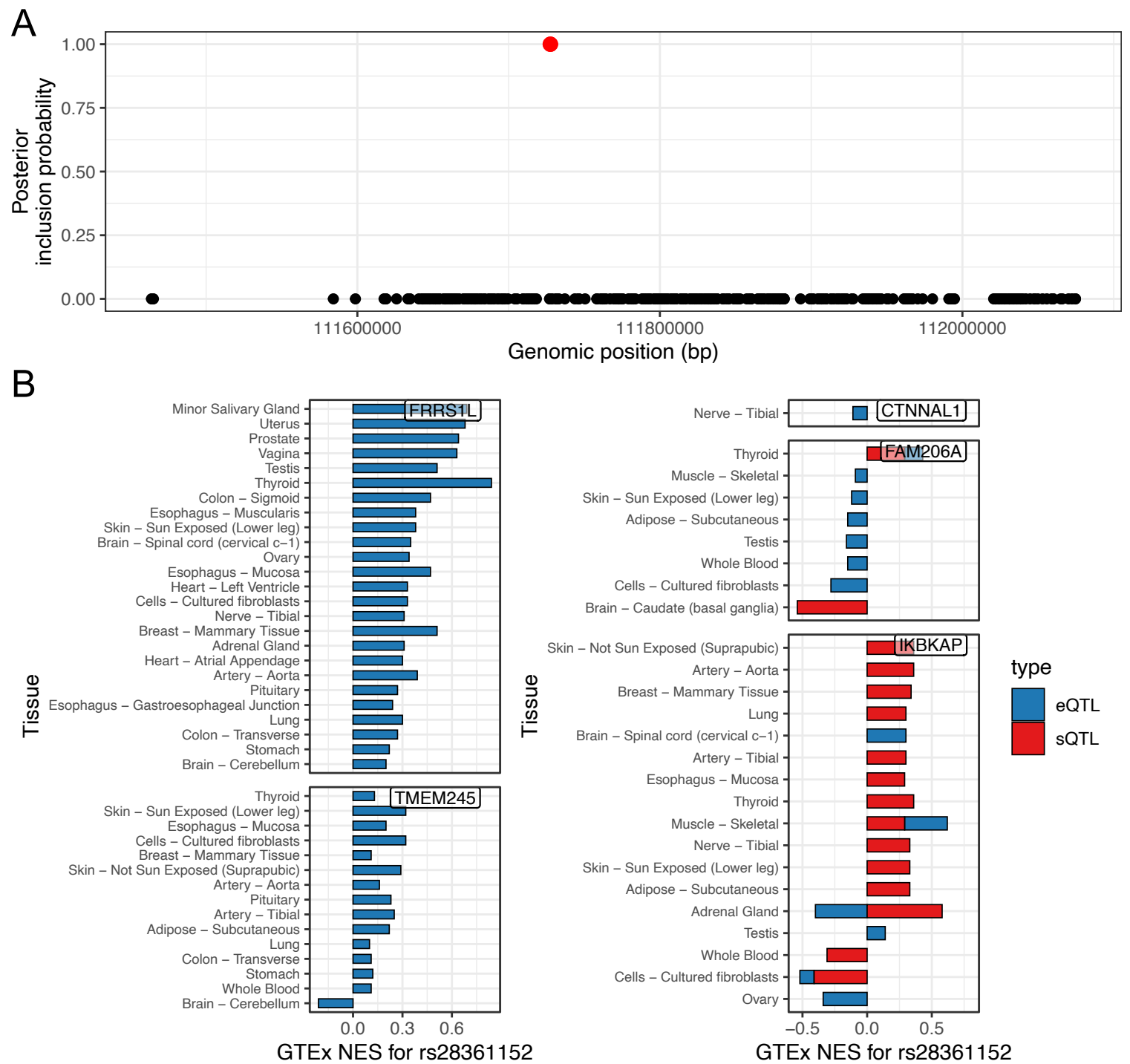

Figure S6

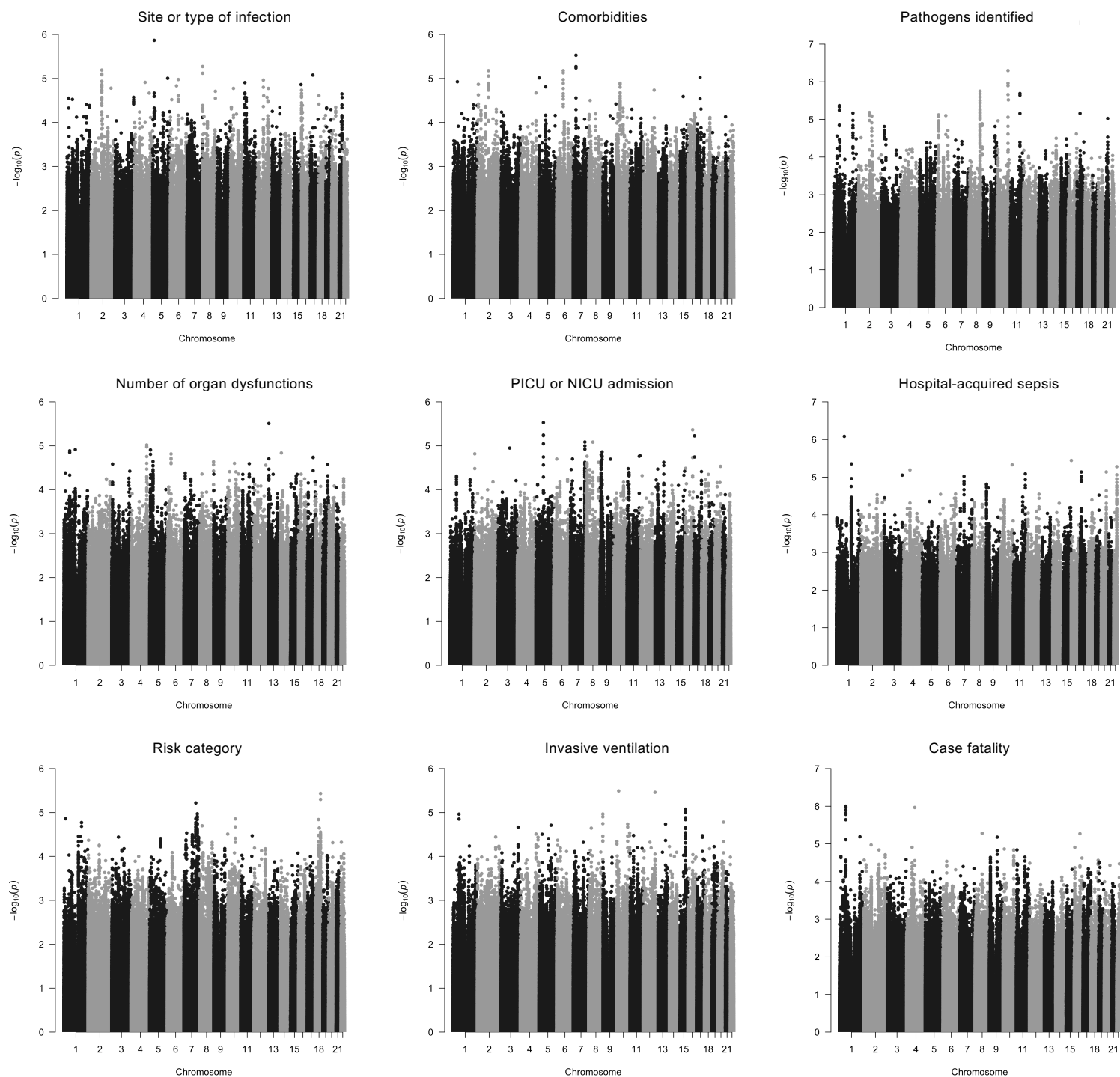

Figure S7

Supplement: Supplementary Figures [file mmc1.pdf]
