# Supplementary material for: Genome-wide association study of paediatric bacteraemia and sepsis
Source: eBioMedicine. 2026 Jun 4;129:106320. doi: 10.1016/j.ebiom.2026.106320 (PMC13266212; doi:10.1016/j.ebiom.2026.106320)
Supplement: Supplementary Captions [file mmc3.docx]

**Supplemental**

**Table S1: SNP quality control filters.**

Samples were genotyped using Illumina OmniExpressExome-8 v1.4 genotyping array, phased with (SHAPEIT2) and imputed (IMPUTE2) using the 1000 Genomes Project phase 3 reference panel. Filtering included missing genotype call rate 10%, minor allele frequencies < 0.05, control HWE 1e^-6^, and subject independence by identify-by-state (IBS). Geno, genotype call rate; HWE, Hardy-Weinberg Equilibrium; MAF, minor allele frequency; LD, linkage disequilibrium.

**Table S2: Association model summary**.

Covariates were included to account for age, sex, study sites. Ancestry was accounted for by GRM. Case-only analysis was also performed for key criteria of clinical characteristics that may be used to assess sepsis.

**Table S3. List of protein-altering variants in LD with lead SNP**

Genomic coordinates are based on the GRCh 37 build. LD estimates are based on the HRC reference panel. Haploreg v4 was used to assess the protein-altering functional annotation of the proxy SNPs.

**Table S4. Extended list of all variants in LD with lead SNP**

The extended list of variants in LD with the lead SNP contain 1519 variants, with ranges in their MAF 0.1-0.5; D’ 0.1-1.0; and R^2^ 0.0-1.0.

**Table S5. eQTL associations for associated SNPs in GTEx v8**

Genome wide significant SNPs in sepsis susceptibility case-control analysis were used to query for eQTL associations in GTEx v8. Results for the lead SNP rs28361152 are shown. P-values included are below the gene-level significant threshold of variant-gene pairs. Genomic coordinates are based on the GRCh 38 build. NES, normalized effect size; sQTL, splicing Quantitative Trait Loci.

**Table S6. sQTL associations for associated SNPs in GTEx v8**

Genome wide significant SNPs in sepsis susceptibility case-control analysis were used to query for eQTL associations in GTEx v8. Results for the lead SNP rs28361152 are shown. P-values included are below the gene-level significant threshold of variant-gene pairs. Genomic coordinates are based on the GRCh 38 build. eQTL, expression Quantitative Trait Loci; NES, normalized effect size.

**Table S7. Statistical summary of demographic and clinical variables**

For each variable, descriptive group comparisons and corresponding P values are reported. These tests are provided to document potential imbalances that might represent confounding factors but do not constitute hypothesis driven analyses.

**Figure S1: Patient cohort characteristics.**

(A) categorical and (B) continuous variables for patient cohort. Our cohort consisted of 650 cases after QC (quality control). Color scale low (blue) to high (yellow).

**Figure S2: Imputation quality.**

The information score is plotted for illustration of a randomly selected region of 5e^6^ SNPs. High quality imputation is present for variants with scores above 0.7-0.8 as shown for (A) total count and (B) cumulative sum. INFO score >0.8 was used for analysis.

**Figure S3: Principal component analysis.**

(A) The Swiss Pediatric Sepsis Study cohort is predominantly made up of individuals of European ancestry (83%, **Table 1**). Within-cohort association was tested for all samples with eight eigenvalues included as quantitative covariates to control for population structure in a mixed model. (B) For case-control analysis population structure required pruning to European only ancestry. The first twenty PCs are shown in (C).

**Figure S4: Gene expression in candidate gene locus**

Gene expression heatmap from 5 genes of interest on Chr 9 in association with sepsis. Annotated for biological context based on Functional Mapping and Annotation of Genome-Wide Association Studies (FUMA). Significantly associated SNPs overlap *ELP1, FAM206A*, and *CTNNAL1* and all five genes were identified as expression Quantitative Trait Loci.

**Figure S5. Regional association and LD diagnostics at the FAM206A / CTNNAL1 locus on chromosome 9**

(A) Regional association plot showing −log₁₀(P) for variants in the locus, coloured by SuSiE credible set and sized by posterior inclusion probability (PIP). A single, narrow peak centred at 111.6–111.7 Mb defines the lead SNP. (B) Diagnostic plot comparing each variant’s z-score ratio (zᵢ/z_lead) against the reference correlation (r) to the lead SNP. Points lie below the diagonal, indicating that correlation in the Swiss cohort is weaker than predicted by the 1000G EUR reference panel, particularly for variants to the right of the peak. (C) Raw association (blue) versus conditional association (red) after removing the expected contribution of the lead variant using reference LD. The conditional curve flattens across the region, showing that the association can be explained by a single signal; the small residual rise at 111.7–111.8 Mb reflects LD mismatch in the reference rather than a second association. (D) Cross-population LD to the lead variant, plotted as r² by position for 1000G populations. European panels show extended LD up to 111.8 Mb, while African panels show rapid decay. Black boxes mark the Swiss association peak (left) and the reference-defined extension (right), illustrating how population LD differences affect locus colouring and conditional behaviour.

**Figure S6. SuSiE fine and GTEx eQTL and sQTL across tissues**

(A) Posterior inclusion probabilities from SuSiE are shown for all variants in the locus. The lead variant rs28361152 had a posterior inclusion probability of 1.0 and all other variants had posterior inclusion probabilities of zero, indicating a single credible signal. (B) Normalised effect sizes from GTEx v8 are plotted for rs28361152 across all tissues with significant regulatory effects. eQTL effects are shown in blue and sQTL effects in red. Effects are grouped by gene and ordered by magnitude within each gene. Gene labels are displayed inside each facet.

**Figure S7. No significant genetic association within case-only characteristics**

Within-cohort (case only) association testing was performed on all 650 case samples. We found no significant association of genetic variants with specific sepsis characteristics. The full model parameters and characteristics are listed in **Table S2**.

***Testing for overlap with an independent mouse model study***

We identified *ELP1* and *CTNNAL1* as candidate genes for susceptibility to paediatric sepsis. These genes (*Elp1* and *Ctnnal1* in mice) were independently reported by Vered et al. (2019) as part of the Collaborative Cross (CC), a systems genetics resource for host-pathogen interaction studies using a multi-parental mouse genetic reference population,

as reviewed by Noll et al.(2019). Vered et al. (2019) challenged 73 pre-CC mouse lines with *Klebsiella pneumoniae* and identified 2 and 6 significantly associated non-coding SNPs in Elp1 and Ctnnal1, respectively. To test whether the overlap of these genes across the two studies exceeded that expected by chance, we applied a weighted Z-test and Fisher’s combined probability test using the reported study sizes (n=1504 for SPSS and n=328 for CC).

First, we used the weighted Z-test, which transforms each study-level P-value into a standard normal deviate and then combines them in proportion to study sample size, thereby giving greater weight to the larger study. Second, we applied Fisher’s combined probability test, which evaluates the joint evidence by summing the logarithms of the individual P-values and comparing the result to the chi-squared distribution with degrees of freedom equal to twice the number of studies. Both approaches assume independence between studies and were implemented in R using the survcomp::combine.test function with the “z.transform” and “fisher” options and the study sample sizes (SPSS n = 1504, CC n = 328).
