# Supplementary material for: Genome-wide association study of paediatric bacteraemia and sepsis
Source: eBioMedicine. 2026 Jun 4;129:106320. doi: 10.1016/j.ebiom.2026.106320 (PMC13266212; doi:10.1016/j.ebiom.2026.106320)
Supplement: Collab Authors [file mmc4.docx]

| **First names** | **Surnames** |
| --- | --- |
| **Christoph** | Aebi |
| **Philipp K. A.** | Agyeman |
| **Walter** | Bär |
| **Christoph** | Berger |
| **Sara** | Bernhard-Stirnemann |
| **Eric** | Giannoni |
| **Ulrich** | Heininger |
| **Christian R.** | Kahlert |
| **Gabriel** | Konetzny |
| **Antonio** | Leone |
| **Giancarlo** | Natalucci |
| **Anita** | Niederer-Loher |
| **Klara M.** | Posfay-Barbe |
| **Christa** | Relly |
| **Thomas** | Riedel |
| **Luregn J.** | Schlapbach |
| **Martin** | Stocker |
